# CLUTTER AS A BARRIER TO MAINTAINING FUNCTIONAL INDEPENDENCE

**DOI:** 10.1093/geroni/igad104.0433

**Published:** 2023-12-21

**Authors:** Mary Dozier, Caitlyn Nix

**Affiliations:** Mississippi State University, Starkville, Mississippi, United States; Mississippi State University, Mississippi State, Mississippi, United States

## Abstract

Excessive levels of household clutter can increase fall risk and subsequently prevent older adults from living independently. This may be a particular concern for rural-dwelling older adults, who are likely to have a stronger sense of connection with their physical location. This talk will present data from a comprehensive baseline assessment administered to older adults enrolled in treatment studies for hoarding disorder in rural Mississippi (n = 25; mean age: 67). The majority of participants described themselves as “collectors,” (79%) and individuals who considered themselves to be collectors were more likely to have experienced at least one intervention from friends, family, or the community (42% vs. 20%). Thirty-two percent of participants reported they had experienced at least one fall in their home in the previous year and 60% reported they were at least “a little bit” concerned about falling in their home, with 20% indicating that they or someone else had expressed concern specifically about clutter increasing their risk of falling. In addition to fall risk, 28% of participants indicated having at least one health hazard in the home (e.g., mold, insect infestation). Forty-three percent of the sample reported low physical functioning and 50% of the sample indicated a high degree of loneliness. These results highlight the impairment experienced by rural-dwelling older adults with high amounts of clutter. Interventions that target de-cluttering may provide additional utility by also increasing the ability of these older adults to age in place.

